# The Effect of COVID-19 on Attendance Rates in Pediatric Residency Training Educational Sessions

**DOI:** 10.7759/cureus.80443

**Published:** 2025-03-11

**Authors:** Trisha Sunderajan, Louisdon Pierre, Noah Kondamudi, Adeyinka Adebayo

**Affiliations:** 1 Pediatric Critical Care Medicine, Loma Linda University Children's Hospital, Loma Linda, USA; 2 Pediatrics, The Brooklyn Hospital Center, Brooklyn, USA; 3 Pediatric Emergency Medicine, The Brooklyn Hospital Center, Brooklyn, USA; 4 Pediatric Critical Care Medicine, The Brooklyn Hospital Center, Brooklyn, USA

**Keywords:** attendance rates, covid-19 impact, medical training, residency education, virtual learning

## Abstract

Introduction: Residency education faced significant challenges during the COVID-19 pandemic. Programs had to adopt new methods to maintain high-quality education while adhering to social distancing restrictions. This necessity prompted medical training programs worldwide to change their learning routines dramatically. A notable shift occurred from traditional classroom lectures to online educational tools, with web-based learning becoming a key strategy to address these challenges.

Objective: This study aimed to evaluate the impact of the transition to virtual learning during the COVID-19 pandemic on attendance rates at scheduled educational sessions in a pediatric residency program.

Hypothesis: We postulated that the introduction of virtual lectures for residency training will increase the attendance of residents.

Methodology: All pediatric residents exposed to in-person and virtual learning during the study period (October 2020) were included. Attendance records were reviewed, and data were stratified based on the year of expected graduation (Class of 2021 and Class of 2022). The attendance rate percent (ARP) was calculated as: (Number of educational sessions attended / Total number of sessions held) × 100. ARP during the prepandemic (traditional method; July-September 2019) period was compared with ARP during the pandemic period (virtual learning; July 2020-September 2020). Statistical analysis was done using chi-square analysis.

Results: A total of 23 residents were subjects in the study: 11 from the graduating class of 2021 and 12 from the graduating class of 2022. In July- October 2019, with 54 lectures, the total expected attendance was 1,242 (23 residents × 54 lectures). In July-October 2020, with 62 educational sessions, the total expected attendance was 1,426 (62 sessions × 23 residents). The actual attendance was 451 (36%) in 2019 compared to 722 (51%) in 2020, a 15% increase (p < 0.00001). This increase was associated with the shift to virtual learning for both classes (p < 0.001).

Conclusion: Our results show a significant increase in educational session attendance rate among our resident group after the implementation of virtual learning, irrespective of their level of training. However, it is not a given that increased attendance is likely to result in increased knowledge and better clinical performance. Virtual learning should be considered a valuable educational tool for residency training.

## Introduction

The COVID-19 pandemic has left its mark on every aspect of resident medical education, influencing every aspect of training and creating new obstacles and opportunities [1]. Many trainees experienced abrupt changes in their daily schedules due to reduced patient volumes, curriculum changes, and the redistribution of hospital staff away from specialty services and toward COVID-19 inpatient units. Social distancing requirements forced many residency programs to rethink the organizational framework of didactic lectures, leading to significant adjustments in how educational content was delivered [2]. This included transitioning from in-person to remote learning, utilizing synchronous (live-streamed) and asynchronous (prerecorded) formats. Our program used Zoom and Webex to continue medical education and lectures, including morning reports, grand rounds, journal club, case management, and morbidity and mortality conferences.

While the future implications of the COVID-19 pandemic on residency education remain uncertain, the situation's urgency has driven many institutions to adopt novel techniques for didactic lectures, focusing on online learning. In this study, we aimed to evaluate the impact of virtual learning on residents’ attendance at scheduled educational sessions during the peak of COVID-19.

We hypothesized that the use of online learning tools may positively influence attendance at educational sessions in our community pediatric residency program. Virtual learning’s accessibility may enhance resident participation in educational activities compared to traditional teaching methods. This study can offer an educational framework that can be useful to other departments in navigating similar challenges during these unprecedented times [3].

The abstract was presented at the American Academy of Pediatrics 2022 session held on February 23, 2022.

## Materials and methods

Study overview

We conducted a single-center retrospective cohort study at a community (city-level) pediatric residency program with 34 residents. Online educational sessions became available in July 2020. The study included 23 pediatric residents: 11 from the graduating class of 2021 and 12 from the graduating class of 2022. These residents attended traditional in-person educational sessions in 2019 (pre-COVID-19 pandemic) and participated in virtual educational sessions introduced in May 2020 during the pandemic. We excluded 11 residents from the graduating class of 2020, who were in their final year in 2019 and only experienced traditional teaching, and 11 residents from the class of 2023, who were first-year residents in 2020 and were solely exposed to online educational sessions. We made these exclusions to maintain uniformity in sampling and enable a valid comparison between the cohorts that experienced both prepandemic in-person sessions and the online learning sessions implemented during the pandemic. This study was approved by the Institutional Review Board at the Brooklyn Hospital Center (approval number 1622184-1).

Outcome measure

We measured the primary outcome by analyzing changes in the attendance percentage rate (APR). The chief residents collected attendance records maintained by the program, which were reviewed. We calculated APR using the formula: (Number of educational sessions attended / Total number of sessions held) × 100. We then compared attendance rates from the prepandemic period (traditional classroom method, July-September 2019) with those from the pandemic period (virtual learning, July-September 2020).

The included residents were categorized into two groups based on their graduation year: the graduating class of 2022 formed the first group, while the graduating class of 2021 formed the second group. We then calculated the percentage increase in attendance during the study period (July-September) and compared it with the same three-month period in 2020.

Data collection

The pediatric department at our institution consists of 34 residents: 11 in the first year, 12 in the second year, and 11 in the third year. Our intern class joined at the beginning of the academic year on July 1, 2020. Due to social distancing rules imposed by the COVID-19 pandemic, all in-person lectures were paused from March to May 2020. The curriculum shifted to remote learning via Zoom and WebEx in May 2020. To maintain consistency, we collected attendance data for the months of July to September 2020 (the COVID-19 period). We compared it to the attendance rates for in-person lectures during the same period in 2019 (pre-COVID-19 periods). Chief residents recorded attendance on a password-protected Excel sheet (Microsoft Corporation, Redmond, WA) in their third postgraduate year.

Statistical analysis

Statistical analysis was carried out using Statistical Package for the Social Sciences, version 28 (IBM Corp., Armonk, NY). Given the qualitative nature of the data, the chi-square test was used to evaluate the increase in attendance rate percentage (ARP). Statistical significance was defined as a p value of less than 0.05. To calculate the percentage increase, we compared the attendance rates against the expected rate of attendance.

## Results

A total of 23 residents met the study criteria: 11 from the graduating class of 2021 and 12 from the graduating class of 2022. In 2019, the study period from July to September included 54 lectures, resulting in an expected total attendance of 1,242 (23 residents × 54 lectures). During the same period in 2020, there were 62 educational sessions, with a total expected attendance of 1,426 (62 sessions × 23 residents).

The cumulative attendance in 2019 was 451, reflecting an attendance rate of 36%. In 2020, the cumulative attendance increased to 722, corresponding to an attendance rate of 51%, which represents a 15% improvement. This increase in attendance rate was statistically significant, with a p value of <0.00001 (Table 1).

**Table 1:** Cumulative attendance of pediatric residents (2019-2020) in a community hospital. These findings show a statistically significant increase in attendance in 2020 after the introduction of virtual learning with a p value of <0.0001

Attendance	2019, n (%)	2020, n (%)	Total
Yes	451 (36)	722 (51)	1,173
No	791 (64)	704 (49)	1,495
Total	1,242	1,426	2,668

For the graduating class of 2021, the total attendance during the 2019 study period was 220. With 54 educational sessions, the expected attendance for the 11 pediatric residents was 594 (54 sessions × 11 residents). In 2020, total attendance increased to 382, while the expected attendance for the 11 residents, based on 62 sessions, was 682 (62 sessions × 11 residents). The ARP was 37% in 2019 compared to 56% in 2020. This increase in attendance was statistically significant, with a p value of <0.00001 (Table 2).

**Table 2:** Attendance for the graduating class 2021 of pediatric residents in a community hospital. These results show a statistically significant increase in attendance in 2020 after the introduction of virtual learning with a p value of <0.001

Attendance	2019, n (%)	2020, n (%)	Total
Yes	220 (37)	382 (56)	602
No	374 (63)	300 (44)	674
Total	594	682	1,276

For the graduating class of 2022, the total attendance during the 2019 study period was 231. With 54 educational sessions conducted, the expected attendance for the 12 residents was 648 (54 sessions × 12 residents). In 2020, the total attendance increased to 340, while the expected attendance for the 12 residents, based on 62 sessions, was 744 (62 sessions × 12 residents). The ARP for 2019 was 36%, compared to 46% in 2020. This increase in attendance was statistically significant, with a p value of 0.000178 (Table 3).

**Table 3:** Attendance for the graduating class of 2022 pediatric residents in a community hospital. These results show a statistically significant increase in attendance in 2020 after the introduction of virtual learning with a p value of <0.001

Attendance	2019, n (%)	2020, n (%)	Total
Yes	231 (36)	340 (46)	571
No	417 (64)	404 (54)	821
Total	648	744	1,392

## Discussion

To our knowledge, this is the first study to meticulously examine how virtual learning has influenced a pediatric residency program. Pediatric residency poses unique challenges, requiring a tailored approach to educational engagement. It demands away rotations in the neonatal intensive care unit (NICU), pediatric intensive care unit, and emergency room (ER), where unpredictable schedules and high-intensity training often make traditional didactics difficult to attend. The shift to virtual learning has the potential to redefine how pediatric residents engage with education, bridging gaps that were once seen as unavoidable. Our three-month comparison over two years revealed increased attendance for online lectures compared to in-person lectures. The convenience and broad reach of virtual lectures largely contributed to this increase. These results align with other studies showing that convenience significantly influences trainees' and faculty members' attitudes toward remote learning [1,4].

Reich and White emphasized that declining admission rates for common pediatric conditions, such as bronchiolitis and asthma, during the COVID-19 pandemic reduced learning opportunities for pediatric residents [5,6]. This finding underlines the need to enhance the accessibility of educational tools to ensure that residents do not miss critical learning experiences during their training.

Our analysis demonstrated a significant increase in attendance at remote lectures across all training levels, highlighting the effectiveness of virtual learning in improving participation. In our program, first-year residents spend three months in the emergency room and two months in the nursery and NICU-rotations that require continuous presence and previously limited attendance at in-person lectures. To ensure comprehensive education, pediatric residents must attend various didactic sessions, including morning reports, grand rounds, journal club, case management, and morbidity and mortality conferences. With the introduction of online learning, residents on ER, NICU, and nursery rotations, though exempt from in-person lectures, were given the option to participate remotely, ensuring continued access to educational content. Additionally, post-call residents are expected to attend prescheduled sessions, and virtual learning has facilitated their participation, allowing them to remain engaged despite demanding schedules.

The COVID-19 pandemic prompted a paradigm shift in the mindset of both educators and trainees [7]. This shift created a valuable opportunity to reassess and refine the core components of medical education while integrating the most effective elements from various educational approaches [8]. Virtual learning also fostered greater collaboration between institutions, allowing educators and leaders to exchange ideas more effectively. Blankenburg et al. highlighted how collaboration through the Association of Pediatric Program Directors can address educational and administrative challenges [9]. This collaboration demonstrated the potential to implement a "flipped classroom" model, where fellows and residents from different institutions share knowledge and learn from one another, promoting the widespread dissemination of information.

The limitations of the study are its single-center cohort design at a community hospital, which may limit generalizability to larger university-based residency programs. Additionally, faculty and resident perspectives on the adoption of and attitudes toward remote learning were not assessed, nor were variations in lecture quality evaluated.

## Conclusions

In conclusion, our data provide preliminary evidence that the convenience of remote learning has increased resident attendance at didactic lectures. Remote learning can be a foundation for future organizational reforms, such as developing hybrid lecture formats and popularizing the flipped classroom model. However, our study did not directly assess the attitudes of residents and faculty toward remote learning or examine how the new educational model affected annual in-service training examination scores and academic performance. These areas could be explored in future research. We hope that additional scientific studies will guide pediatric and other specialty training programs in navigating the uncertainties of residency education in the post-COVID-19 era.
